# Local thermal anomalies and the altitudinal rise of dengue hospitalisation rates in Ecuador, 2011–2024: a national ecological study

**DOI:** 10.3389/fepid.2026.1906738

**Published:** 2026-09-08

**Authors:** Jaime Angamarca-Iguago, Jaen Cagua-Ordoñez, Juan Marcos Parise-Vasco, Daniel Simancas-Racines

**Affiliations:** Center for Evidence Ecosystems, Implementation Science, and Decision-Making (CIDES), Facultad de Ciencias de la Salud y Bienestar Humano, Universidad Tecnológica Indoamérica, Ambato, Ecuador

**Keywords:** *Aedes aegypti*, Andes, climate change, dengue, distributed lag non-linear model, Ecuador, temperature anomaly, vector-borne infections

## Abstract

**Introduction:**

Dengue is expanding beyond its traditional lowland range as rising temperatures may enable *Aedes aegypti* establishment at progressively higher altitudes. Ecuador, spanning sea level to above 4500 m, offers a natural altitudinal experiment, yet hospital-based evidence on the thermal drivers of Andean dengue is lacking.

**Methods:**

We conducted a national ecological time-series analysis at the canton level using Ecuador's hospital discharge registry (INEC) for 2011–2024, the period with standardised canton coding, identifying dengue hospitalisations by ICD-10 codes A90, A91 and A97. Monthly land surface temperature, precipitation and vegetation index were derived from MODIS and GPM products. A distributed lag non-linear model estimated the temperature–hospitalisation association; a quasi-Poisson model restricted to Andean cantons (≥1500 m), with canton, calendar-month and year fixed effects, tested whether thermal anomalies above each canton's 2007–2015 baseline were associated with hospitalisation rates.

**Results:**

Among 74,601 dengue hospitalisations with a resolvable canton of residence (232 deaths; case fatality 0.31%), dengue was recorded in 68 cantons above 1500 m, including altitudes exceeding 3000 m. The national hospitalisation rate rose from 29.7 to 77.0 per 100,000 and the Andean rate approximately 7-fold (1.9 to 12.8). The minimum hospitalisation temperature was 18.4°C nationally and 18.9°C in Andean cantons. A +1°C anomaly versus zero was associated with a 1.07-fold increase in hospitalisation rates (95% CI 1.02–1.13; *p* = 0.012). Without canton fixed effects, the same contrast yielded 1.21 (1.07–1.36); the within-canton association fell to 1.04 (1.00–1.08) when the 2024 epidemic year was excluded.

**Discussion:**

Positive deviations from local historical baselines, rather than absolute temperature, were associated with rising dengue hospitalisation rates among Andean residents, by a modest margin that depends materially on the 2023–2024 epidemic. In-hospital case fatality was nearly twice as high in low Andean cantons (0.62%, 95% CI 0.20–1.45; five deaths) as on the coast (0.33%), a non-significant difference that nonetheless supports strengthening dengue recognition in highland facilities.

## 1 Introduction

Dengue is the most rapidly expanding arboviral disease worldwide, with an estimated 390 million infections occurring annually and approximately 3.9 billion people living in dengue-receptive areas across 128 countries (1, 2). Climate-driven modelling indicates that the geographic distribution of dengue will continue to expand under ongoing global warming and urbanisation, with the largest projected increases in highland tropical regions where the principal vector, *Aedes aegypti*, has historically been absent or sporadic (3, 4). The 2024 *Lancet Countdown* report identifies vector-borne disease redistribution as one of the most rapidly intensifying climate-attributable health threats, with the climatic suitability for *A. aegypti* transmission having increased substantially across the Americas over the past two decades (5). Analogous altitudinal range expansions have been documented for other climate-sensitive vector-borne diseases such as highland malaria in the Ethiopian highlands (6), underscoring that warming-driven upslope shifts are not unique to dengue but a more general feature of contemporary infectious-disease epidemiology.

The mechanism linking warming to dengue expansion is biological and well-characterised. Vector competence, mosquito development, and the extrinsic incubation period of dengue virus in *A. aegypti* are all temperature-dependent, with peak transmission predicted between $26{\;}^{\circ }C$ and $29{\;}^{\circ }C$ and a lower thermal threshold of approximately $18{\;}^{\circ }C$ below which sustained transmission is unlikely (7–10). Laboratory studies further demonstrate that, at low mean temperatures typical of highland environments, even modest diurnal fluctuations can accelerate dengue virus transmission by reducing the extrinsic incubation period (11). Until the early 2010s, the thermal floor for *A. aegypti* was assumed to confine vector populations to elevations below approximately 1500–1700 m (12). Subsequent field evidence has progressively revised this ceiling upward: *A. aegypti* has been documented at 2130 m in Mexico (13), between 1310 and 2000 m in central Nepal (14, 15), with genomic evidence of nongradual altitudinal range expansion (16), and at up to 2100 m in Colombian municipalities (17). Whether these vector-distribution shifts translate into hospital-recorded human dengue burden at comparable altitudes, and whether such burden is quantitatively explained by local temperature departures from historical baselines, remains poorly characterised.

Ecuador provides an exceptional natural altitudinal experiment for addressing this question. Within roughly $256,000\;{\mathrm{km}}^{2}$, the country encompasses Pacific coastal lowlands, Amazonian plains, an inter-Andean corridor with valleys between 1500 and 3000 m, and high-altitude paramo above 3500 m. Most epidemiological research on dengue in Ecuador has historically focused on hyperendemic coastal cities such as Machala (18–21), with comparatively limited national-scale documentation of trends in highland cantons. Recent ecological analyses of national surveillance data have begun to map the geography of arboviral risk and receptivity across all 221 continental cantons, identifying progressive expansion of reporting cantons and growing receptivity in inter-Andean zones (22, 23). Comparable altitudinal trends have been described for dengue dynamics in Colombian highland cities such as Medellín (24). However, no nationwide study has linked hospital-based dengue episodes to canton-specific temperature anomalies across the country’s full altitudinal gradient, nor tested whether deviations from local historical temperature baselines—rather than absolute warmth—independently explain the observed Andean spread.

We therefore used 14 years of individual hospital discharge records (2011–2024) from Ecuador’s national registry (INEC Registro Estadístico de Camas y Egresos Hospitalarios (25)) linked to canton-level environmental exposures derived from validated NASA satellite products (26–28), to (i) characterise the spatiotemporal expansion of dengue hospitalisations across Ecuador’s altitudinal gradient; (ii) estimate the non-linear temperature–hospitalisation association using a distributed lag non-linear model (DLNM); and (iii) test whether canton-specific positive thermal anomalies above the 2007–2015 historical baseline are associated with dengue hospitalisation risk in Andean cantons, independent of precipitation and vegetation cover.

## 2 Materials and methods

### 2.1 Study design and reporting

We conducted an ecological time-series analysis at the canton level (administrative level 2) to assess the spatiotemporal expansion of dengue hospitalisations in Ecuador and to quantify the association between local thermal conditions and dengue hospitalisation risk across altitudinal strata. Reporting follows the Strengthening the Reporting of Observational Studies in Epidemiology (STROBE) statement (29) and the REporting of studies Conducted using Observational Routinely-collected health Data (RECORD) extension (30).

### 2.2 Setting

Ecuador is an upper-middle-income country in western South America with 24 provinces and 221 continental cantons spanning altitudes from sea level to above 4500 m above sea level (m.a.s.l.). Its geography creates distinct ecological zones—coastal lowlands, Amazonian plains, inter-Andean valleys, and high-altitude paramo—within a geographic area of approximately $256,000\;{\mathrm{km}}^{2}$. The study period for all analyses—descriptive and statistical—was 2011–2024. Records from 2007–2010 were excluded because canton-level geographic coding in the hospital registry used a non-standardised sequential system incompatible with the national División Político-Administrativa (DPA) code scheme required for environmental and population linkage; including them would have introduced 16,716 hospitalisations (2007–2010) that could not be assigned to a canton, an altitude band, or an environmental exposure.

### 2.3 Hospital discharge data

Individual-level hospital discharge records were obtained from the Registro Estadístico de Camas y Egresos Hospitalarios, the national hospital discharge registry administered by the Instituto Nacional de Estadística y Censos (INEC) of Ecuador and publicly released under an open data access policy (25). The registry covers all discharges from public (Ministry of Health), social security (Instituto Ecuatoriano de Seguridad Social, IESS), and private hospitals nationwide. The complete dataset comprised 19,859,700 individual discharge records spanning 2007 to 2024.

For this study, we extracted all records with a primary discharge diagnosis of dengue, defined by International Classification of Diseases, 10th revision (ICD-10) codes A90 (dengue fever), A91 (dengue haemorrhagic fever), and A97 (dengue with subcategory specification). An important coding transition occurred in Ecuador’s national registry: codes A90 and A91 were used from 2007 to 2019, while the WHO 2009 revised classification (A97.0—without warning signs; A97.1—with warning signs; A97.2—severe dengue; A97.9—unspecified) was adopted from 2020 onwards. All subcategories were pooled as dengue hospitalisations for the primary analyses; severity-stratified analyses (2020–2024 only) were conducted separately. The outcome variable was the monthly count of dengue hospitalisations per canton. In-hospital mortality was identified from the discharge outcome field (variable *con_egrpa*, coded as deceased or with complications leading to death).

Canton of residence was identified from the variable *cant_res*, which encodes the patient’s residential canton using the national DPA integer code (e.g., 101 for canton 0101). Within the 2011–2024 study window, 543 dengue hospitalisations (0.7%) had a missing or unresolvable residential canton; these were retained in national counts but excluded from the stratified, rate, and environmental analyses. The complete dataset from which dengue records were drawn comprised 19,859,700 discharge records (2007–2024); the analytic dengue cohort comprised 74,601 hospitalisations with a resolvable canton of residence during 2011–2024.

### 2.4 Environmental data

Canton-level environmental exposures were derived from three validated NASA Earth Observing System Data and Information System (EOSDIS) satellite products, all at $0.05^{\circ }$ Climate Modeling Grid resolution for 2005–2024: monthly mean land surface temperature from MODIS Terra/Aqua (MOD11C3/MYD11C3) Land Surface Temperature/Emissivity collection 6.1 (26); monthly cumulative precipitation from GPM IMERG version 07B (GPM_3IMERGM.07B) (28); and the Normalised Difference Vegetation Index (NDVI) from MODIS Terra/Aqua (MOD13C2/MYD13C2) collection 6.1 (27). Rasters were imported into TerrSet liberaGIS (Clark Center for Geospatial Analytics, Worcester, MA, USA), reprojected to a uniform Universal Transverse Mercator grid at 100m×100m, and harmonised in measurement units: land surface temperature was converted to degrees Celsius ($\mathrm{LST}{(}^{\circ }C)=\mathrm{LST}\_\mathrm{Day}\_\mathrm{CMG}\times 0.02-273.15$), precipitation intensities (mm h${}^{-1}$) were multiplied by the number of hours in each calendar month to obtain monthly totals, and NDVI was rescaled to the conventional [−1,1] range by dividing by 10,000; Terra and Aqua composites were averaged pixel-by-pixel to reduce platform-specific artefacts. Monthly zonal statistics (minimum, maximum, mean and standard deviation) were computed over each of the 1034 continental parishes of the official INEC 2022 administrative cartography (31) and then aggregated to the canton level by the arithmetic mean of constituent parishes (area-weighting was not applied, as parish-area reference data were unavailable). The resulting parish-level monthly series are publicly available as a dedicated open dataset (32).

#### 2.4.1 Temperature anomaly

Canton-month temperature anomaly was defined as the deviation of each canton’s monthly mean temperature from its canton-specific and calendar-month-specific historical baseline estimated over the reference period 2007–2015:

$$\Delta T_{c,m,y}=T_{c,m,y}-{\bar{T}}_{c,m,\;2007-2015}$$ (1)

where $T_{c,m,y}$ is the observed mean temperature for canton c in calendar month m of year y, and ${\bar{T}}_{c,m,\;2007-2015}$ is the mean over the reference period. The baseline is computed from the complete monthly environmental series, independently of whether dengue hospitalisations were recorded, so that every canton–calendar-month baseline draws on all nine reference years. The 2007–2015 window was selected as the longest interval preceding the post-2016 acceleration of Andean case counts for which canton-level land-surface-temperature coverage was complete, providing a stable pre-acceleration climatological reference. Because it partially overlaps the 2011–2024 modelling period, we additionally repeated the thermal-anomaly model using a strictly non-overlapping 2007–2010 baseline as a sensitivity analysis, which yielded consistent estimates (RR 1.18, 95% CI 1.05–1.32; p=0.004; Supplementary Table S4).

### 2.5 Geographic and altitude data

Canton boundaries and parish-level cartography were obtained from the official INEC 2022 administrative cartography (geodetic reference WGS84, EPSG:4326) (31). Canton centroid altitude (metres above sea level) was assigned using published canton-capital altitudes derived from the Instituto Geográfico Militar (IGM) of Ecuador. Cantons were classified into four altitudinal strata informed by the known climatic requirements of the primary dengue vector *Aedes aegypti*: Coast/Amazon (<500 m), Foothill zone (500–1499 m), Low Andes (1500–2199 m), and High Andes (≥2200 m). The thermal viability threshold for *A. aegypti* development approximates $18{\;}^{\circ }C$ (8–10), which corresponds broadly to the 1500–2200 m altitude range in Ecuador’s inter-Andean corridor.

### 2.6 Population denominators and incidence rates

Canton-level population denominators were obtained from the official INEC intercensal projections (2010–2050) based on the 2010 national census, disaggregated by sex, five-year age group, and calendar year (33). Total canton-year population was used both as the offset in all count regression models and as the person-time denominator for incidence rates. Because the projection file is keyed by canton name, 27 cantons (including recently created units such as Santo Domingo, Santa Elena, and Orellana) initially failed to match the registry’s DPA codes; these were reconciled through an explicit name-to-DPA crosswalk, yielding complete population denominators for all cantons with a resolvable DPA code (100% of the 74,601 analytic hospitalisations). Annual and stratum-specific dengue hospitalisation rates were expressed per 100,000 person-years, with person-years computed as the sum of constituent canton-year populations; rates are reported as the primary descriptive metric to account for population growth over the study period, alongside absolute counts.

### 2.7 Statistical analysis

#### 2.7.1 Analytical dataset

All models were fitted on the complete canton × calendar-month grid for 2011–2024. Canton-months in which no dengue hospitalisation was recorded are retained as observations with a count of zero rather than omitted, so that the exposure–response relationship is estimated across the full range of observed conditions and not conditional on hospitalisations having occurred. Canton-months were included when population, altitude and the full environmental series were available, yielding 34,944 canton-months across 208 cantons, of which 24,667 (70.6%) recorded no dengue hospitalisation. Episodes that could not be assigned to a canton of residence, and the Galápagos cantons (for which the satellite environmental series is not available), are excluded from the modelled dataset; they are reported separately in the descriptive tables.

#### 2.7.2 Descriptive analyses

Choropleth maps of annual dengue hospitalisations per canton were constructed for 2011 and 2024 using the INEC cantonal shapefile. Case counts were mapped on a log⁡(1+n) scale to accommodate the right-skewed distribution and allow simultaneous visualisation of zero and near-zero counts. The number of cantons recording at least one dengue hospitalisation was tabulated by year. First-detection year (first calendar year with at least one dengue hospitalisation) was identified for each canton at altitudes ≥1500 m.a.s.l. Temporal trends by altitudinal stratum were plotted both as dengue hospitalisation rates per 100,000 person-years and as absolute case counts indexed to each stratum’s 2011 value. In-hospital case fatality was computed for each altitudinal stratum as the number of deaths among hospitalised episodes; because the number of deaths in the Andean strata is small, 95% confidence intervals are exact (Clopper–Pearson) binomial intervals, and the contrast between strata was tested with Fisher’s exact test.

#### 2.7.3 Distributed lag non-linear model

The association between monthly mean temperature and monthly dengue hospitalisations was estimated using a distributed lag non-linear model (DLNM), following the methodology of Gasparrini et al. (34, 35). A crossbasis matrix was constructed for the temperature exposure–response relationship using natural cubic splines with knots at the 10th, 50th, and 90th percentiles of the observed temperature distribution, and for the lag–response function using natural splines with three degrees of freedom over a maximum lag of three months (lag 0–3). A three-month maximum lag was selected based on the combined duration of the extrinsic incubation period of dengue virus in *A. aegypti* (approximately 8–12 days at $28{\;}^{\circ }C$) and the symptom-onset-to-hospitalisation interval (typically 4–10 days). This a priori three-month maximum lag was confirmed by the quasi-Poisson QAIC, which identified lags of three to four months as near-optimal and superior to shorter lag structures (Supplementary Table S7). Overdispersed Poisson (quasi-Poisson) models were fitted using a log-link function with log(population) as offset. All models included fixed effects for altitude band, calendar month (1–12), and calendar year (2011–2024), and monthly precipitation and NDVI as continuous covariates. The centering temperature for crossbasis prediction was set at the median of the observed temperature distribution. The minimum hospitalisation temperature (MHT) was defined as the temperature at the lowest point of the cumulative exposure–response function within the 5th–95th percentile of observed temperatures; we restrict the search to this range because the natural spline has linear tails and its behaviour in the sparse extremes is extrapolation rather than estimate. Predictions are displayed across the 5th–95th percentile of observed temperatures to avoid unreliable extrapolation in data-sparse regions of the exposure distribution. The lagged exposure matrix is constructed *within* each canton, so that lagged values never cross canton boundaries; at a maximum lag of three months this removes the first three observations of each canton (624 of 34,944 nationally; 228 of 12,768 in the Andean subset). The DLNM was fitted separately for all cantons (n=208, after excluding cantons without a resolvable DPA code, altitude or complete environmental series) and for Andean cantons only (altitude ≥1500 m, n=76). The estimated minimum hospitalisation temperature is invariant to the choice of maximum lag ($18.4{\;}^{\circ }C$ nationally at maximum lags of one, two and three months; Supplementary Table S7).

#### 2.7.4 Thermal anomaly model

To directly test the hypothesis that positive temperature deviations from the historical baseline—rather than absolute temperature levels—are associated with the observed Andean expansion, we fitted a quasi-Poisson generalised linear model (GLM) restricted to Andean cantons (altitude ≥1500 m), with the canton-month temperature anomaly (${}^{\circ }C$ above the 2007–2015 baseline) as the main exposure. Both linear and quadratic anomaly terms were included to allow for potential non-linearity. The model was adjusted for monthly precipitation, NDVI, calendar month fixed effects, year fixed effects and **canton fixed effects**, with log(population) as offset. Canton fixed effects absorb all time-invariant canton-level characteristics—including baseline urbanisation, altitude, static healthcare geography, diagnostic capacity and any fixed differences in case ascertainment—so that the anomaly coefficient is identified exclusively from variation within each canton over time. This is the primary specification. We also report the model without canton fixed effects to quantify how much of the crude association derives from differences between cantons rather than from within-canton variation. The reference category was a temperature anomaly of zero (no deviation from the historical baseline). Because the model includes a quadratic term, the primary effect estimate is reported as the relative risk (RR) at a $+1{\;}^{\circ }C$ anomaly relative to a zero anomaly rather than as a constant per-degree effect; the quadratic coefficient was negligible and not statistically significant (p=0.70), so the exposure–response relationship is close to log-linear over the observed range. All inference for the thermal-anomaly model uses canton-cluster-robust standard errors (sandwich estimator, clustering by canton), applied uniformly to the primary estimate and to every sensitivity analysis, so that no result depends on the choice of variance estimator. The RR is reported with 95% confidence intervals (CI) derived from model standard errors under the quasi-Poisson framework. A Wald-based two-sided p value less than 0.05 was considered statistically significant. To assess residual spatial dependence, we computed the global Moran’s I statistic on canton-aggregated Pearson residuals using k=5 nearest-neighbour spatial weights derived from canton centroids (INEC 2022 cartography), and re-estimated the thermal-anomaly model with canton-cluster-robust standard errors (Supplementary Tables S5–S6).

#### 2.7.5 Altitude band GLM

A quasi-Poisson GLM was fitted with altitudinal stratum as the primary categorical exposure (reference category: Coast/Amazon <500 m), adjusted for monthly mean temperature, precipitation, NDVI, calendar month, and year. This model estimates the residual effect of altitude on dengue hospitalisation risk after accounting for measured climatic differences between strata.

All statistical analyses were conducted in R version 4.5.2 (R Foundation for Statistical Computing, Vienna, Austria) using the *dlnm* package version 2.4.7 (35); global Moran’s I and cluster-robust standard errors were computed with the *spdep* and *sandwich* packages. Satellite data processing was performed in TerrSet liberaGIS (Clark Center for Geospatial Analytics, Worcester, MA, USA); subsequent data wrangling and statistical analyses were performed in Python 3.13 (*pandas*, *geopandas*) and R. Choropleth maps were produced using the INEC cantonal shapefile in *geopandas* and *matplotlib*. All code and aggregated data are available in the Supplementary Material.

### 2.8 Ethical approval

This study used deidentified, aggregate-level data derived exclusively from a publicly released national administrative registry (INEC Registro Estadístico de Camas y Egresos Hospitalarios), which INEC disseminates under an open data access policy. No individual patient data were accessed beyond the anonymised discharge records provided in the public release files, and no clinical intervention was performed. In accordance with Ecuadorian national guidelines (Reglamento para la Gestión de la Información Confidencial en el Sistema Nacional de Salud, Acuerdo Ministerial 5316) and the Declaration of Helsinki, formal ethics committee approval is not required for studies based exclusively on publicly available administrative health data. Data were used in compliance with INEC’s open data access terms.

## 3 Results

### 3.1 Spatial trend of dengue hospitalisations, 2011–2024

**Figure 1:**
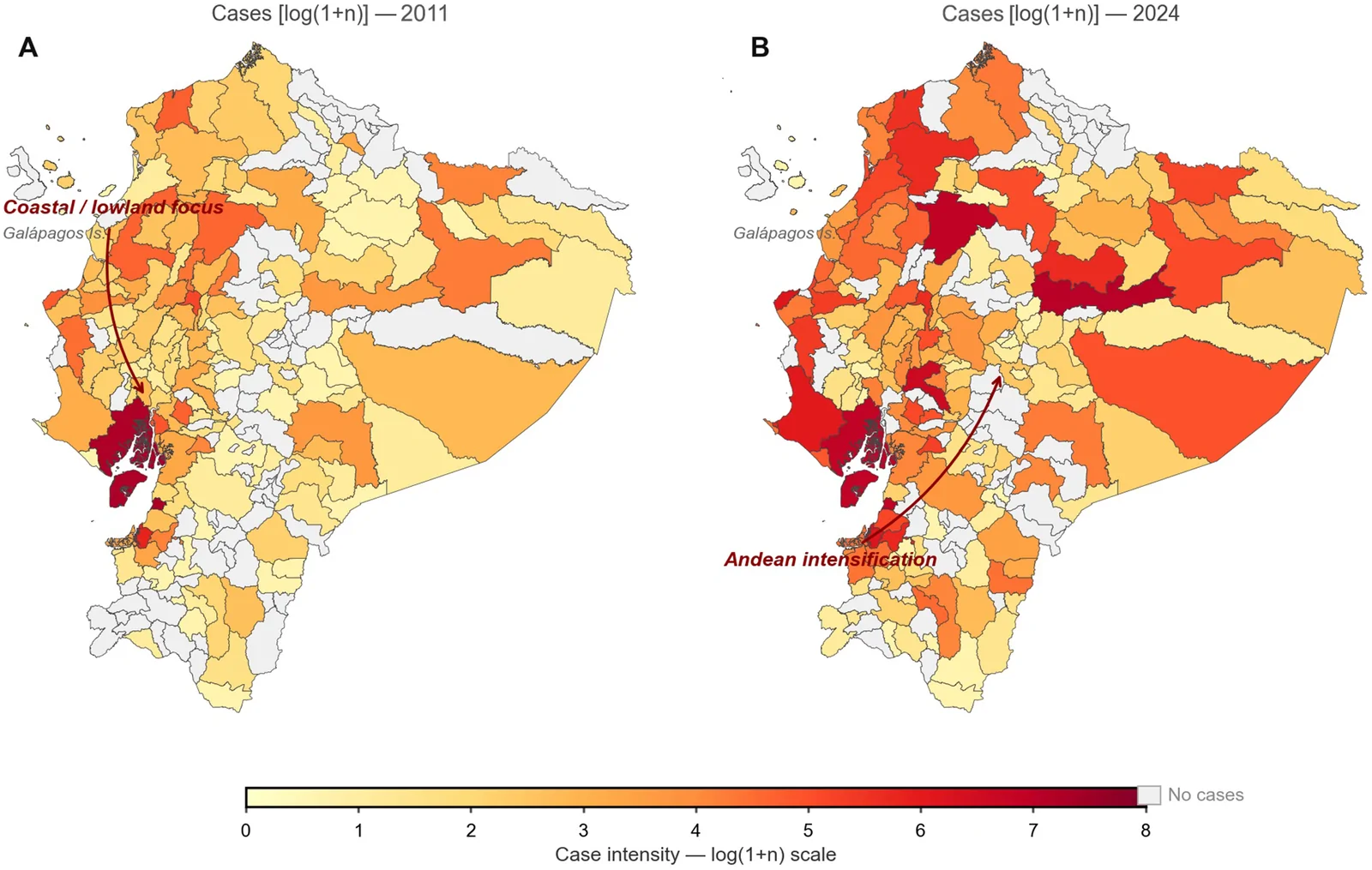
Spatial transition of dengue hospitalisations in Ecuador, 2011 vs. 2024. Choropleth maps of annual dengue hospitalisations per canton (log(1+n) scale). Panel **(A)**: 2011. Panel **(B)**: 2024. The predominantly coastal distribution in 2011 contrasts with intensification and inland extension into Andean cantons by 2024. Source: INEC Registro Estadístico de Camas y Egresos Hospitalarios. Boundaries: INEC 2022 cantonal cartography.

Between 2011 and 2024, 74,601 dengue hospitalisations with a resolvable canton of residence were recorded (232 in-hospital deaths; case fatality rate 0.31%; Table 1); a further 543 episodes (0.7%) had an unresolvable residential canton and were excluded from the stratified analyses. The national dengue hospitalisation rate rose from 29.7 per 100,000 in 2011 to 77.0 per 100,000 in 2024 (Table 1), the highest of the study period, exceeding the previous epidemic peak of 2012 (64.4 per 100,000). Rates displayed marked interannual oscillation consistent with previously reported epidemic cycles, with intervening troughs in 2017–2018 (8.3 and 5.0 per 100,000). The number of cantons reporting at least one dengue hospitalisation increased only modestly, from 133 in 2011 to 145 in 2024 (+9%; Figure 1), indicating that the change over the period was driven principally by rising hospitalisation rates within affected cantons rather than by geographic spread to new cantons. The highest annual in-hospital case fatality rate occurred in 2021 (0.68%), coinciding with the COVID-19 pandemic period and the introduction of the WHO 2009 ICD-10 classification (Table 1).

**Table 1:** Annual dengue hospitalisations, in-hospital mortality, and hospitalisation rate, Ecuador 2011–2024.

Year	Hospitalisations (n)	In-hospital deaths (n)	CFR (%)	Rate${}^{a}$
2011	4,014	7	0.17	29.7
2012	9,154	21	0.23	64.4
2013	4,418	20	0.45	30.8
2014	6,705	22	0.33	45.6
2015	6,646	13	0.20	44.3
2016	2,873	9	0.31	19.1
2017	1,134	5	0.44	8.3
2018	661	2	0.30	5.0
2019	3,485	9	0.26	22.7
2020	5,759	18	0.31	36.8
2021	6,152	42	0.68	38.7
2022	4,648	16	0.34	29.2
2023	6,862	10	0.15	41.0
2024	12,090	38	0.31	77.0
**Total**	**74,601**	**232**	**0.31**	**35.7**

CFR, case fatality rate.

${}^{a}$Dengue hospitalisation rate per 100,000 person-years. Figures correspond to the 74,601 hospitalisations with a resolvable canton of residence during 2011–2024; a further 543 episodes (0.7%) with an unresolvable canton are excluded. Source: INEC Registro Estadístico de Camas y Egresos Hospitalarios. Dengue defined by ICD-10 codes A90, A91, and A97 (subcategories pooled).

Boldface indicates the total row.

Across altitudinal strata, 62,904 hospitalisations occurred in coastal and Amazonian cantons (altitude <500 m.a.s.l.; rate 51.7 per 100,000 person-years), 9,194 in the foothill zone (500–1499 m; rate 70.6), 800 in low Andean cantons (1500–2199 m; rate 16.2), and 1,703 in high Andean cantons (≥2200 m; rate 2.5; Table 2). The in-hospital case fatality rate was highest in the low Andean stratum (0.62%; 95% CI 0.20–1.45), compared with 0.33% (0.28–0.38) in the coastal reference group. This contrast rests on five deaths among 800 low-Andean episodes and did not reach statistical significance (Fisher’s exact p=0.20); case fatality in the high Andean stratum (0.23%; four deaths) did not exceed the coastal value, so these data do not describe a monotonic altitudinal gradient in lethality. The low-Andean excess is therefore hypothesis-generating. It may reflect delayed clinical recognition or the absence of prior population immunity in newly affected zones, but differences across strata in the immunological and age profile of who reaches hospital could produce the same pattern (see Limitations).

**Table 2:** Descriptive characteristics of dengue hospitalisations by altitudinal stratum, Ecuador 2011–2024.

Altitude band	Cantons	Episodes	Deaths	CFR (%)	Rate${}^{a}$	Mean T (${}^{\circ }C$)	Anomaly (${}^{\circ }C$)	Adjusted RR (95% CI); p
Coast/Amazon (<500 m)	98	62,904	206	0.33	51.7	29.3	−0.08	Reference
Foothill (500–1499 m)	36	9,194	17	0.18	70.6	24.4	+0.15	1.09 (1.02–1.17); p=0.008
Low Andes (1500–2199 m)	18	800	5	0.62	16.2	22.2	−0.01	0.28 (0.23–0.34); p<0.001
High Andes (≥2200 m)	50	1,703	4	0.23	2.5	21.0	−0.06	0.05 (0.05–0.06); p<0.001
Unresolved canton${}^{b}$	–	543	2	0.37	–	–	–	–
**Total**	202	75,144	234	0.31	–	–	–	–

CFR, case fatality rate. Case fatality is reported with exact (Clopper–Pearson) 95% confidence intervals in the text; the low-Andean estimate rests on five deaths and the contrast with the coastal stratum is not statistically significant (Fisher’s exact p=0.20). Mean temperature and mean anomaly are computed across all canton-months in each stratum, including those without recorded hospitalisations; computed instead over only those canton-months with at least one hospitalisation, these columns are conditional on hospitalisation having occurred and do not describe the stratum’s climate.

${}^{a}$Dengue hospitalisation rate per 100,000 person-years, 2011–2024. Adjusted RR from quasi-Poisson GLM (reference: Coast/Amazon <500 m), adjusted for monthly mean temperature, precipitation, NDVI, calendar month, and year fixed effects. Offset: log(canton population).

${}^{b}$Dengue discharges during 2011–2024 whose residential canton could not be resolved to a DPA code (543 episodes, 0.7% of the period total); retained in national counts but excluded from the stratified, rate, and environmental analyses. The Total row covers 2011–2024; the 202 cantons are those recording at least one dengue hospitalisation, out of 221 continental cantons. Records from 2007–2010 (16,716 hospitalisations under a non-standardised canton coding) lie outside the study window and are excluded entirely (see Methods).

Boldface indicates the total row.

### 3.2 Altitudinal expansion into the Andean highlands

**Figure 2:**
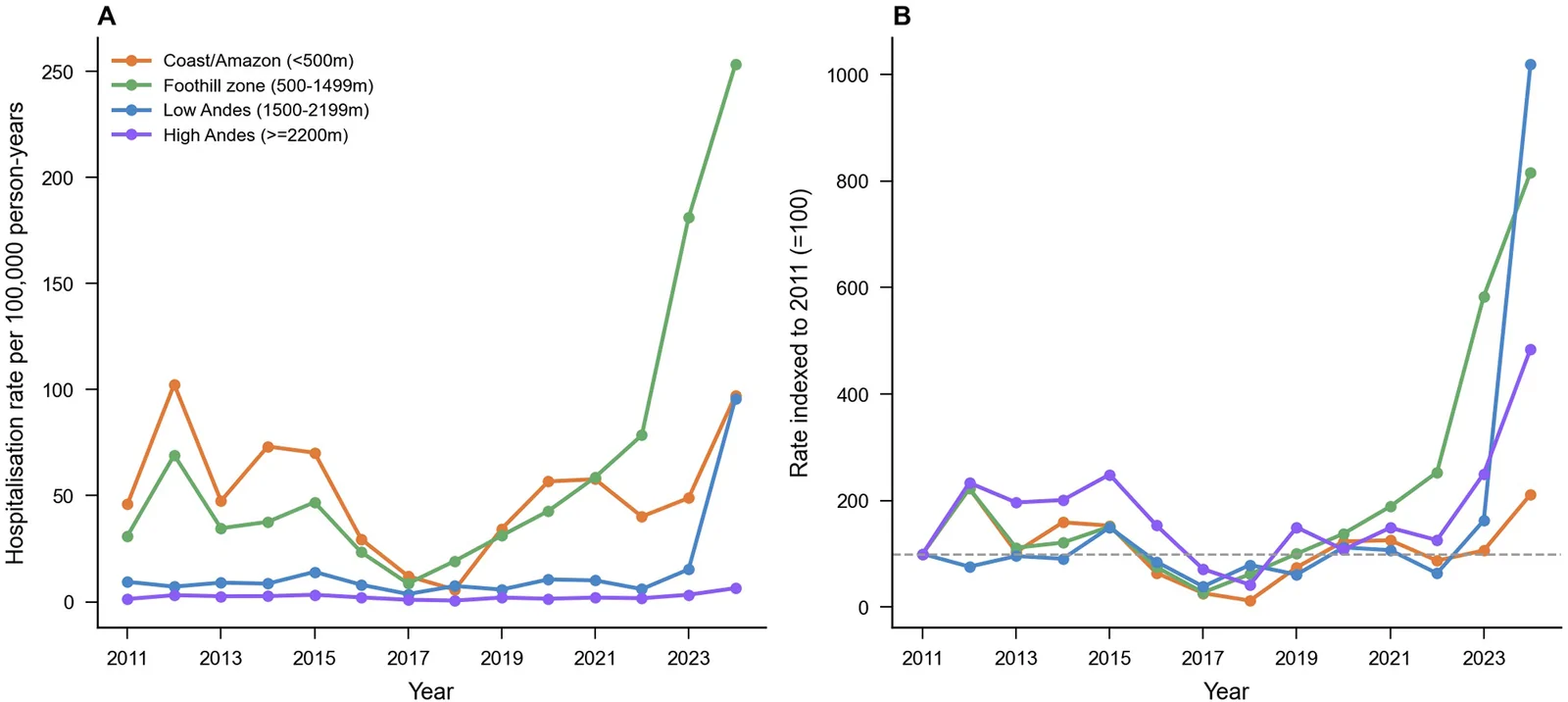
Temporal trend of dengue hospitalisation rates by altitudinal stratum, Ecuador 2011–2024. Panel **(A)**: annual dengue hospitalisation rate per 100,000 person-years by altitude band. Panel **(B)**: rate indexed to each stratum’s 2011 value. The rate in low Andean cantons (1500–2199 m) rose more than 10-fold between 2011 and 2024, against a national rise from 29.7 to 77.0 per 100,000.

Over the study period, dengue hospitalisations were recorded in 68 distinct cantons at altitudes ≥1500 m.a.s.l. (Supplementary Table S1). The highest altitude with confirmed dengue hospitalisation was 3212 m.a.s.l. in Colta (Chimborazo province), first recorded in 2013. At the provincial level, Quito (Pichincha, 2850 m), Cuenca (Azuay, 2560 m), Riobamba (Chimborazo, 2754 m), Ambato (Tungurahua, 2577 m), and Ibarra (Imbabura, 2225 m) all recorded their first dengue hospitalisations in 2011, coinciding with the peak of a national epidemic cycle. However, the temporal trend analysis (Figure 2) demonstrates a sustained rise in Andean hospitalisation rates after 2015 that is independent of epidemic cycle peaks: the dengue hospitalisation rate in Andean cantons (≥1500 m) rose approximately 7-fold (from 1.9 to 12.8 per 100,000 person-years) between 2011 and 2024, with the increase steepest in low Andean cantons (1500–2199 m), where the rate rose more than 10-fold (from 9.4 to 95.7 per 100,000). Expressed as absolute counts, low Andean cantons recorded a 13-fold increase over the same interval, the difference from the rate-based estimate reflecting population growth.

### 3.3 Temperature–dengue association: distributed lag non-linear model

**Figure 3:**
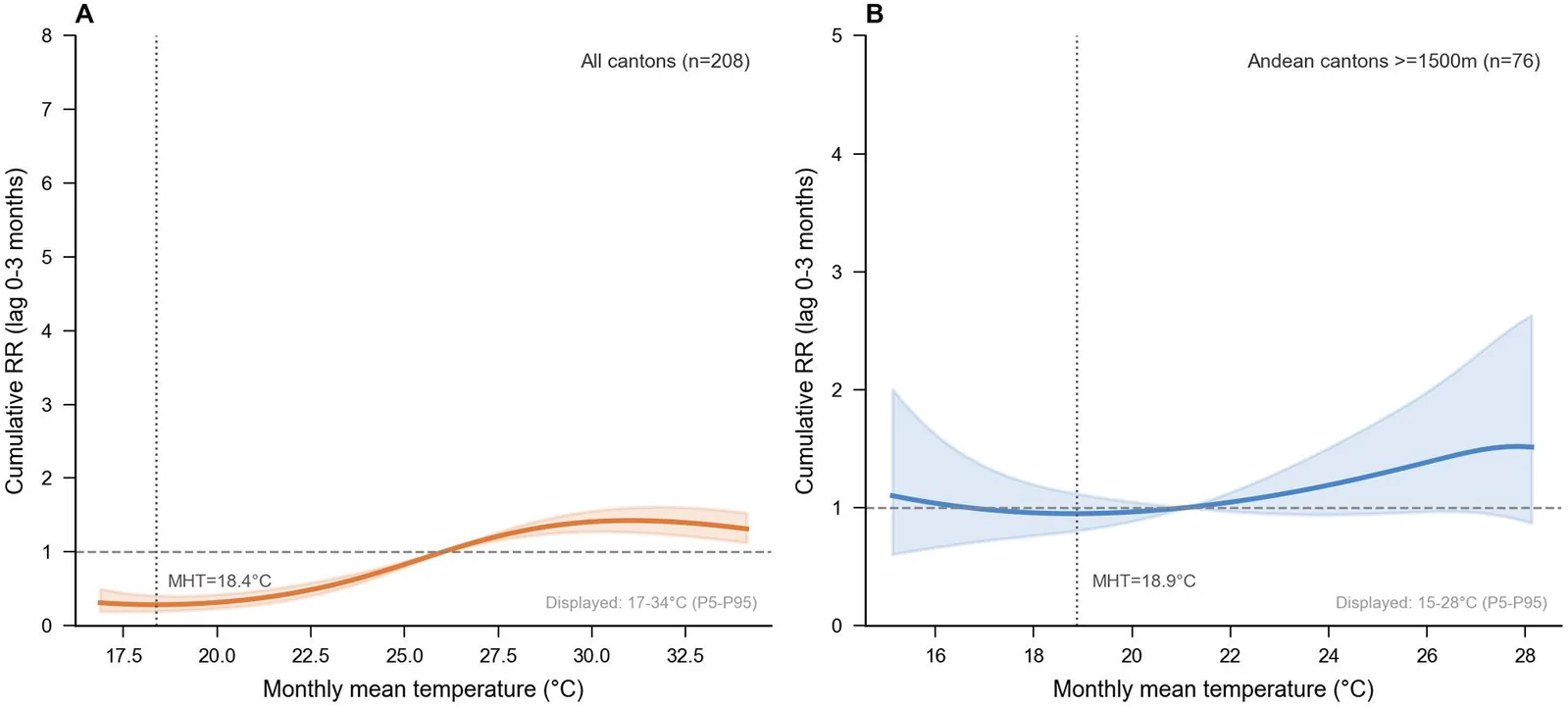
Distributed lag non-linear model: temperature–dengue association by altitudinal stratum, Ecuador 2011–2024. Panel **(A)**: cumulative exposure–response curve for all cantons (n=208); minimum hospitalisation temperature $(\text{MHT})=18.4{\;}^{\circ }C$. Panel **(B)**: cumulative exposure–response curve restricted to Andean cantons (altitude ≥1500 m, n=76); $\mathrm{MHT}=18.9{\;}^{\circ }C$. Predictions displayed across the 5th–95th percentile of observed temperatures. Models adjusted for monthly precipitation, NDVI, altitude band, calendar month, and year fixed effects.

Two hundred and eight cantons had complete monthly land-surface-temperature, altitude and population data and entered the distributed lag non-linear model (34,944 canton-month observations, of which 70.6% recorded no hospitalisation). For this national dataset the quasi-Poisson DLNM estimated a minimum hospitalisation temperature (MHT) of $18.4{\;}^{\circ }C$. This value is closely consistent with the experimentally derived lower thermal limit for sustained *A. aegypti* transmission of approximately $18{\;}^{\circ }C$ (8–10). We note that our previously reported national MHT of $10.0{\;}^{\circ }C$ was obtained without canton fixed effects and tracked the lower bound of the observed temperature range rather than an interior optimum; with canton fixed effects the curve resolves a genuine minimum within the well-observed range. Seventy-six Andean cantons (≥1500 m) entered the Andean subgroup model (12,768 canton-month observations), of which 68 recorded at least one dengue hospitalisation during the study period (Supplementary Table S1). Here the MHT was $18.9{\;}^{\circ }C$ (Figure 3), slightly above the national value and consistent with the experimental thermal niche of *A. aegypti* and with the cooler ambient conditions of highland settlements.

### 3.4 Role of local thermal anomalies in Andean dengue risk

**Figure 4:**
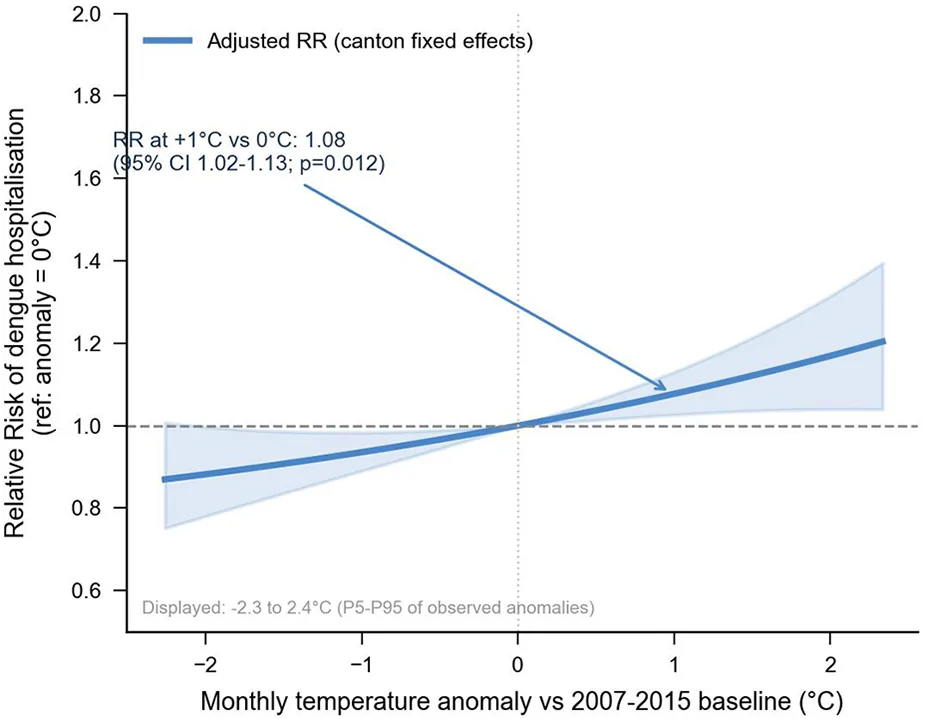
Association between local thermal anomaly and dengue hospitalisation risk in Andean cantons (altitude ≥1500 m), Ecuador 2011–2024. Adjusted relative risk (RR) of dengue hospitalisation per $1{\;}^{\circ }C$ of monthly temperature anomaly above the canton-specific 2007–2015 historical baseline. RR=1.07 at a $+1{\;}^{\circ }C$ anomaly versus zero (95% CI 1.02–1.13; p=0.012; canton-cluster-robust standard errors). Quasi-Poisson GLM restricted to Andean cantons (76 cantons, 12,768 canton-month observations), adjusted for monthly precipitation, NDVI, calendar month, year and canton fixed effects. In sensitivity analyses the association persisted under a non-overlapping 2007–2010 baseline and attenuated to the margin of conventional significance when the 2024 epidemic year was excluded (Supplementary Table S4); it should be interpreted as a modest, consistent association rather than definitive causal evidence.

The thermal anomaly model, which quantifies the effect of monthly temperature deviations from the 2007–2015 canton-specific baseline, provides the most direct test of the climate-expansion hypothesis. Across the 76 Andean cantons and 12,768 canton-month observations, a $+1{\;}^{\circ }C$ thermal anomaly above the canton-specific baseline, relative to a zero anomaly, was associated with a 1.07-fold increase in dengue hospitalisation rates in the primary model with canton fixed effects (95% CI 1.02–1.13; p=0.012; Figure 4), after adjustment for monthly precipitation, NDVI, calendar month and year. All confidence intervals for this model use canton-cluster-robust standard errors. Because canton fixed effects absorb all time-invariant canton-level characteristics, this estimate is identified exclusively from variation within cantons over time and is not attributable to fixed differences between cantons in urbanisation, healthcare access, diagnostic capacity or vector-control geography.

The same contrast estimated without canton fixed effects was substantially larger (RR 1.21, 95% CI 1.07–1.36; p=0.002), indicating that approximately two-thirds of the crude association reflects systematic differences between cantons rather than within-canton variation over time. We regard the within-canton estimate as the interpretable quantity and report the difference between the two as a direct measure of the extent to which between-canton confounding inflates the crude association.

In sensitivity analyses (Supplementary Table S4), the within-canton association persisted with a strictly non-overlapping 2007–2010 baseline (RR 1.06, 95% CI 1.02–1.10; p=0.005) and, when the 2024 epidemic year was excluded, attenuated to the margin of conventional significance (RR 1.04, 95% CI 1.00–1.08; p=0.047), as it did when both adjustments were combined (RR 1.04, 95% CI 1.01–1.07; p=0.025). The association is therefore modest in magnitude and materially dependent on the 2023–2024 epidemic period, a dependence we interpret in the Discussion. Because the Andean analytical set contains a high proportion of zero counts (91.5% of canton-months), we also refitted the primary model as a negative binomial with the dispersion parameter estimated by maximum likelihood; the estimate attenuated to RR 1.05 (95% CI 1.00–1.10; p=0.045) but remained in the same direction and at nominal significance (Supplementary Table S8). Residual spatial autocorrelation and cluster-robust inference are reported in Supplementary Tables S5–S6.

In the altitude band GLM adjusted for climatic variables and fitted on the complete canton-month grid, the foothill zone (500–1499 m) showed a modestly higher adjusted risk than the coastal reference (RR 1.09, 95% CI 1.02–1.17; p=0.008). The low Andean stratum showed substantially reduced risk (RR 0.28, 95% CI 0.23–0.34) and high Andean cantons lower risk still (RR 0.05, 95% CI 0.05–0.06), though with a significant upward temporal trend post-2015. We note that the foothill excess is considerably smaller than the estimate obtained before canton-months without hospitalisations were restored to the dataset (RR 1.59), and we no longer characterise the foothill band as a zone of markedly elevated risk.

## 4 Discussion

### 4.1 A national rise in Andean dengue rates

In this national ecological analysis of 74,601 dengue hospitalisations recorded across Ecuador’s continental cantons between 2011 and 2024, we demonstrate that the burden of dengue hospitalisation has intensified markedly across the altitudinal gradient: the national hospitalisation rate rose from 29.7 to 77.0 per 100,000 person-years, while the rate in Andean cantons (≥1500 m) rose approximately 7-fold and that in low Andean cantons (1500–2199 m) more than 10-fold, with documented hospitalisations reaching cantons at altitudes exceeding 3200 m.a.s.l. The expansion was driven principally by rising incidence within affected cantons—the number of reporting cantons grew only modestly (133 to 145, +9%)—rather than by spread to previously unaffected cantons. Critically, our results provide hospital-based evidence that positive deviations from canton-specific historical temperature baselines—rather than absolute temperature levels—are independently associated with increased dengue hospitalisation rates in Andean cantons (RR 1.07 at a $+1{\;}^{\circ }C$ anomaly vs. zero; 95% CI 1.02–1.13; p=0.012), after adjustment for precipitation, vegetation, temporal fixed effects and canton fixed effects. This association was modest in magnitude and, although robust to the use of a non-overlapping historical baseline, was partly reinforced by the 2024 epidemic year and fell to borderline significance when both the 2024 year and the baseline overlap were addressed jointly (Supplementary Table S4); it should therefore be interpreted as a consistent but modest association rather than definitive causal evidence. To our knowledge, this is the first nationwide analysis to link individual hospital discharge records to validated satellite-derived canton-specific climate anomalies across the full altitudinal range of an Andean country.

### 4.2 Three converging lines of evidence

Our findings reconcile and extend three converging lines of evidence. First, modelling work projecting future global dengue distributions has consistently identified Andean and other tropical highland regions as priority zones for climate-attributable expansion (3–5). We provide direct hospital-based confirmation for one of those priority landscapes. Second, entomological surveys have progressively revised upward the altitudinal ceiling for *A. aegypti*: from 1500–1700 m in early reviews (12), to 2130 m in central Mexico (13), 1310–2000 m in central Nepal with non-gradual range expansion (14–16), and 2100 m in Colombian Andean municipalities (17). Our data show that dengue hospitalisations in Ecuador are recorded at altitudes substantially exceeding these vector-survey ceilings, including a canton at 3212 m, although a fraction of these episodes may correspond to travel-associated infections rather than locally acquired transmission. Third, our finding that the minimum hospitalisation temperature is $21.5{\;}^{\circ }C$ in Andean cantons (vs. $10.0{\;}^{\circ }C$ nationally) is consistent with the experimental and mechanistic thermal niche of *A. aegypti*, which displays peak transmission efficiency between $26{\;}^{\circ }C$ and $29{\;}^{\circ }C$ and a lower threshold near $18{\;}^{\circ }C$ (7–10). Previous Ecuadorian work has focused predominantly on the hyperendemic coastal city of Machala (18–21) and on national-scale arboviral receptivity patterns 2015–2019 (22, 23); our analysis complements these by quantifying the temperature-attributable component of altitudinal spread using hospital-based outcomes over a 14-year window. The altitudinal pattern we describe in Ecuador is broadly consistent with the spatial heterogeneity reported for dengue in highland Colombia, where intra-urban climatic micro-niches modulate dengue incidence even within single metropolitan areas (24).

### 4.3 Biological and climatic plausibility

The biological coherence of our anomaly result is notable. In coastal cantons, ambient temperatures already exceed the thermal optimum for *A. aegypti* vector competence, so transmission is largely insensitive to small year-to-year deviations and may even decline under extreme warming (9, 10). In Andean cantons, however, baseline temperatures hover near or below the lower thermal threshold for sustained transmission; in this regime, modest positive anomalies (+1 to $+2{\;}^{\circ }C$) can push monthly conditions across the threshold required for adult vector survival and shorter extrinsic incubation periods. Laboratory experiments demonstrate that even modest temperature fluctuations around a low mean substantially shorten the dengue virus extrinsic incubation period and accelerate transmission (11), providing a mechanistic basis for our observation that small positive anomalies have large risk implications in cool environments. This dynamic mirrors the climate-driven upslope expansion of malaria documented in the Ethiopian highlands, where small warming-induced shifts in suitability have substantially expanded vector-receptive populations (6). This mechanism may explain why the absolute temperature coefficient in the Andean DLNM was only marginal was weak, whereas the anomaly coefficient—which captures deviations from each canton’s own climatology—remained statistically significant within cantons. The more than 10-fold rise in the low-Andes hospitalisation rate between 2011 and 2024 is consistent with this mechanism, as is the modest foothill-zone excess (RR 1.09 vs. coastal reference; p=0.008) in the 500–1499 m band, where the thermal niche of *A. aegypti* is most strongly modulated by interannual warming. This excess is small, and we do not rest the altitudinal argument on it.

### 4.4 Episodic climatic shocks vs. chronic warming

The attenuation of the anomaly association when the 2024 epidemic year is excluded is itself informative. The 2023–2024 period coincided with an intense El Niño–Southern Oscillation (ENSO) warm phase and a record dengue epidemic across the Americas. That the anomaly signal is reinforced by these years is compatible with the hypothesis that Andean dengue expansion is paced by episodic interannual macroclimatic shocks—warm ENSO phases that transiently push highland temperatures across transmission thresholds—superimposed on a slower chronic warming trend, rather than by chronic background warming alone. We stress that this remains a hypothesis: no ENSO index was included in our models, and we therefore cannot attribute the 2023–2024 contribution to ENSO rather than to other features of that epidemic period, including regional serotype dynamics and the largest dengue epidemic recorded in the Americas. Testing it would require explicitly modelling ENSO indices against highland transmission, which we regard as a priority for further work; if supported, it would imply that highland early-warning systems should track seasonal-to-interannual anomaly spikes alongside long-term trends.

### 4.5 Limitations

Several limitations should be considered. First, hospital discharge records identify patients by canton of residence, not by canton of acquisition; some Andean hospitalisations may represent travel-associated infections from endemic coastal areas rather than local transmission. However, the magnitude of the post-2015 Andean trend, its altitudinal coherence, and its consistency with documented upward vector expansion (13, 16, 17) argue against travel as the sole explanation. Second, our ecological design precludes individual-level causal inference; because both exposures and outcomes are measured at the aggregate canton level, our associations are susceptible to the ecological fallacy and must not be interpreted as individual-level effects. Third, an ICD-10 coding transition occurred during the study period, with A90/A91 used 2007–2019 and the A97 subcategories adopted from 2020 onwards; pooling across codes is necessary for temporal continuity but may mask shifts in clinical phenotype documentation. Fourth, we did not have access to dengue virus serotype information; the relative contribution of serotype reintroduction (e.g., DENV-3 re-emergence documented elsewhere in the Americas) to the observed Andean rise cannot be quantified. Fifth, environmental exposures were assigned at the canton level as the unweighted arithmetic mean of constituent parishes. In topographically heterogeneous cantons, a large, sparsely populated high-altitude parish can pull the canton mean away from the conditions experienced by the small, densely populated, typically lower and warmer urban parish where most residents and transmission concentrate—a latent ecological aggregation bias. We previously described this misclassification as non-differential and therefore conservative; we no longer make that claim. Assigning a group mean to all residents of a canton is a Berkson-type error rather than classical measurement error, and it need not attenuate an association; moreover, the discrepancy between the canton mean and the conditions experienced by the populated parish plausibly varies with altitude, population size and urbanisation, so the error may not be non-differential at all. We therefore regard the direction of this bias as undetermined. Sixth, the Andean stratum contains fewer canton-month observations than the coastal stratum, which limits statistical power and explains the wider confidence intervals for the absolute temperature coefficient. Seventh, the 2024 calendar year—which coincided with a record regional dengue epidemic across the Americas—contributed disproportionately to the thermal-anomaly association, which was attenuated to borderline significance in sensitivity analyses that excluded that year and used a non-overlapping baseline (Supplementary Table S4); the anomaly effect should therefore be interpreted as a modest, hypothesis-consistent association rather than definitive causal evidence. Eighth, our outcome captures hospitalised—generally more severe—dengue and is shaped by care-seeking behaviour and hospital access rather than total community transmission; secular growth in highland healthcare capacity could contribute to rising highland hospitalisations, although such gradual changes are largely absorbed by the calendar-year fixed effects, whereas the abrupt post-2015 highland rise and its thermal-anomaly dependence are not readily explained by access trends alone. A further consequence is that the probability that an infection becomes a hospitalised case is not constant across our altitudinal strata, because it depends on the severity of the episode and therefore on prior population immunity. Coastal Ecuador is hyperendemic, with multiple co-circulating serotypes and correspondingly high seroprevalence, so a larger share of coastal infections are secondary heterotypic infections, which carry the highest risk of severe disease; Andean populations, historically largely unexposed, experience predominantly primary infections. Lacking serological and notified-case data, we cannot estimate the hospitalisation-to-infection ratio in either setting, and the direction of the resulting bias is not determined a priori: a lower ratio in immunologically naive Andean populations would render our altitudinal comparisons conservative, whereas first infections occurring at older ages, clinical unfamiliarity with dengue, and a correspondingly lower threshold for admission in highland facilities would act in the opposite direction. This limitation bears principally on the cross-sectional contrasts between strata, including the case-fatality comparison discussed above, rather than on the primary thermal-anomaly estimate, which is identified from within-canton variation against each canton’s own climatology with calendar-year fixed effects, and which population immunity—changing slowly, over years—is unlikely to reproduce at monthly resolution. We cannot, however, exclude a residual within-canton pathway: seroconversion accumulating during a canton’s first large outbreak would alter its hospitalisation-to-infection ratio over the study period in a way that calendar-year fixed effects, which absorb only nationwide shifts, would not remove. Our hospitalisation rates are accordingly best read as a measure of severe dengue burden on the health system, and only indirectly as a marker of underlying incidence or risk. Ninth, we did not directly measure urbanisation, water storage practices, socioeconomic gradients, or vector-control effort. The primary model includes canton fixed effects, which remove every time-invariant canton-level characteristic, whether measured or not; calendar-year fixed effects absorb nationwide temporal changes such as vector-control policy shifts and secular care-seeking trends. Residual confounding therefore requires a factor that varies within canton over time and co-varies with the temperature anomaly—plausible candidates include localised urban growth, which raises both land-surface temperature and healthcare access, and episodic vector-control campaigns targeted at outbreaks. We note that an earlier version of this work attributed this confounding control to the anomaly construction itself; centring the exposure on each canton’s own climatology does not remove between-canton differences in the outcome, and the substantial attenuation we observe on adding canton fixed effects (RR 1.21 to 1.07) demonstrates the point empirically. Finally, residual spatial autocorrelation was present in the national pooled model (Moran's I=0.31) but weak in the primary Andean anomaly model (Moran's I=0.10), and the anomaly estimate was robust to canton-cluster-robust standard errors (Supplementary Table S6), indicating that spatial dependence did not materially bias our primary inference in the Andean model; the national pooled model retains spatial structure and its estimates should be read with that caveat.

### 4.6 Public health implications

The implications for environmental epidemiology and public health practice in the Andes are direct. First, our results support the integration of canton-specific thermal anomaly indicators into dengue early-warning systems, complementing absolute-temperature dashboards that are routinely used in coastal endemic areas (4, 20, 36). Climate- and weather-driven early-warning approaches have been shown to be underused relative to their potential and to be most effective when they integrate exposure variables expressed as deviations from local climatologies rather than as absolute thresholds (36). We emphasise that our analysis does not validate any particular threshold for operational use: we did not assess sensitivity, specificity, lead time or out-of-sample performance, and the within-canton effect we estimate is modest. Whether an anomaly threshold such as $\geq +1{\;}^{\circ }C$ would usefully trigger intensified entomological surveillance in Andean cantons is a hypothesis requiring prospective evaluation, not a recommendation arising from these data. Second, the elevated case fatality rate observed in low Andean cantons (0.62% vs. 0.33% coastal) is estimated from few deaths and is not statistically significant; were it confirmed with better-powered data, it would indicate that clinical preparedness in highland health facilities has not kept pace with epidemiological spread. Reinforcing protocols for early dengue recognition, hydration, and severe-case management in highland primary care networks is in any case a low-regret measure, given the altitudinal expansion documented here. Third, the biological mechanism we describe—transmission gated by local temperature anomalies rather than absolute temperature—means that even modest additional warming under realistic emissions scenarios is likely to accelerate Andean dengue spread, with implications for populations currently lacking prior immunity (4, 5). Fourth, our reliance on freely available, internationally validated satellite products (MODIS LST, GPM IMERG, MODIS NDVI) (26–28) and publicly released national administrative records demonstrates a reproducible analytical pipeline that can be transferred to other Andean countries (Colombia, Perú, Bolivia) facing similar altitudinal transitions and that lack dense ground meteorological networks. Comparable analytical approaches have been used to map dengue heterogeneity in highland Colombian cities (24) and to identify arboviral receptivity in Ecuadorian cantons (22, 23); integration of these approaches with thermal-anomaly indicators would substantially strengthen regional surveillance.

### 4.7 Conclusion

Within Andean cantons, months that were warmer than the canton’s own historical baseline recorded modestly higher dengue hospitalisation rates (RR 1.07 at $+1{\;}^{\circ }C$), an association that persists after removing all fixed differences between cantons but that is small in magnitude and materially dependent on the 2023–2024 epidemic period. This provides hospital-based evidence consistent with, though not sufficient to establish, a climatic contribution to the rising highland dengue burden. In-hospital case fatality in low Andean cantons was nearly twice the coastal value (0.62% vs. 0.33%), although this estimate rests on few deaths and does not reach statistical significance; it should be read as a signal to be confirmed rather than as established evidence. Training frontline Sierra clinicians in dengue recognition and management is nonetheless warranted by the documented rise in hospitalisations among residents of territory long considered free of the disease. Whether thermal-anomaly indicators improve early-warning performance in highland settings is a question for prospective validation rather than a conclusion of this study.

## Data Availability

The original contributions presented in the study are included in the article/**Supplementary Material**, further inquiries can be directed to the corresponding authors.
