# Supplementary material for: Local thermal anomalies and the altitudinal rise of dengue hospitalisation rates in Ecuador, 2011–2024: a national ecological study
Source: Front Epidemiol. 2026 Sep 8;6:1906738. doi: 10.3389/fepid.2026.1906738 (PMC13593839; doi:10.3389/fepid.2026.1906738)
Supplement: Supplementary file 1 [file Datasheet1.pdf]

Supplementary Material

1 SUPPLEMENTARY DATA

This Supplementary Material contains: (i) Table S1 listing all 68 Andean cantons (altitude $\geq$1500 m.a.s.l.) with first calendar year of recorded dengue hospitalisation, (ii) Table S2 with dengue hospitalisations by ICD-10 severity subcategory and altitudinal stratum (2020–2024), (iii) Table S3 with annual dengue hospitalisations, rates, and mean canton-month temperature anomaly in the Andean strata, (iv) Table S4 with sensitivity analyses for the thermal-anomaly association, (v) Table S5 with the residual spatial-autocorrelation (global Moran's I) diagnostics, (vi) Table S6 with cluster-robust inference for the thermal-anomaly model, (vii) Table S7 with the QAIC-based lag-structure selection under canton-wise lagging and the invariance of the minimum hospitalisation temperature to that choice, and (viii) Table S8 with the model-specification sensitivity of the thermal-anomaly estimate. Tables S4–S8 were recomputed on the rebuilt analytical panel (complete canton $\times$ month grid, zero counts retained, baseline drawn from the full environmental series) with canton fixed effects; they were produced with the reproducible scripts in `analysis_R3/` provided with the code.

The rebuilt canton-month panel used for the statistical analyses (`panel_full_rebuilt.csv`), together with the R and Python source code required to reproduce all figures and tables (`build_full.py`, `tables.R`, `curves.R`, `moran.R`, `figs.py`), is provided with the submission and is additionally available from the corresponding author.

2 SUPPLEMENTARY TABLES

Table S1. Andean cantons (altitude $\geq$1500 m.a.s.l.) with year of first recorded dengue hospitalisation in EcoSalud 360 (ICD-10: A90, A91, A97), Ecuador 2011–2024.

Canton	Altitude (m.a.s.l.)	First year detected
Colta	3212	2013
Mejía	3118	2012
Cañar	3104	2011
Mocha	3080	2022
Quero	3080	2017
Guano	3040	2013
Tisaleo	3020	2013
El Tambo	3000	2013
Sigchos	2983	2013
Espejo	2980	2014
Tulcán	2980	2014
Pujilí	2980	2012
Saquisilí	2924	2015
Quito	2850	2011

Canton	Altitude (m.a.s.l.)	First year detected
Cayambe	2830	2011
Montúfar	2823	2016
Santiago de Píllaro	2800	2016
Olmedo	2800	2016
Pucará	2800	2013
Pedro Moncayo	2790	2014
Suscal	2780	2024
Chunchi	2780	2014
Latacunga	2757	2011
Riobamba	2754	2011
Pallatanga	2739	2017
Salcedo	2683	2011
Espíndola	2680	2012
Guaranda	2668	2011
San Pedro de Pelileo	2600	2015
Biblián	2600	2014
Ambato	2577	2011
Rumiñahui	2561	2011
Cuenca	2560	2011
Otavalo	2530	2012
Saraguro	2525	2022
Azogues	2518	2011
Cumandá	2500	2011
Sigsig	2440	2013
San Miguel	2430	2011
Cotacachi	2418	2013
Chillanes	2400	2012
Antonio Ante	2387	2012
San Miguel de Urcuquí	2384	2015
Chordeleg	2348	2014
Alausí	2347	2012
Gualaceo	2327	2013
Guamote	2256	2011
Ibarra	2225	2011
Bolívar	2200	2013
Chimbo	2200	2012
Paute	2179	2013
Gonzanamá	2165	2011
Pimampiro	2165	2013
Girón	2162	2013
Patate	2150	2012
Sevilla de Oro	2070	2012
Loja	2060	2011
Pangua	1900	2011

Canton	Altitude (m.a.s.l.)	First year detected
Chilla	1900	2014
Célica	1900	2015
Archidona	1850	2011
Baños de Agua Santa	1820	2012
Quilanga	1750	2012
Sozoranga	1670	2021
Mira	1650	2020
Atahualpa	1600	2012
Santa Isabel	1580	2011
Calvas	1500	2013

Source: EcoSalud 360 (INEC Anuario de Camas y Egresos Hospitalarios, 2011–2024). First-year detection is defined as the earliest calendar year with at least one dengue hospitalisation recorded for residents of the canton.

Table S2. Dengue hospitalisations by ICD-10 severity subcategory and altitudinal stratum, Ecuador 2020–2024 (period in which the WHO 2009 revised ICD-10 classification was applied).

Altitude band	Severity (ICD-10)	Cases (n)	Deaths (n)
Coast/Amazon ($<$500 m)	A97.0 – Without warning signs	4,746	15
	A97.1 – With warning signs	12,444	27
	A97.2 – Severe dengue	272	23
	A97.9 – Unspecified	10,170	41
Foothill (500–1499 m)	A97.0	735	5
	A97.1	3,229	3
	A97.2	76	1
	A97.9	2,495	3
Low Andes (1500–2199 m)	A97.0	61	1
	A97.1	365	2
	A97.2	9	0
	A97.9	108	1
High Andes ($\geq$2200 m)	A97.0	166	0
	A97.1	114	1
	A97.2	7	0
	A97.9	514	1

Severity codes per the WHO 2009 revised ICD-10 classification adopted by Ecuador's national registry from 2020 onwards. A97.0 = dengue without warning signs; A97.1 = dengue with warning signs; A97.2 = severe dengue; A97.9 = dengue, unspecified.

Table S3. Annual dengue hospitalisations and mean canton-month temperature anomaly in Andean strata (altitude ≥1500 m.a.s.l.), Ecuador 2011–2024.

	Low Andes (1500–2199 m)				High Andes (≥2200 m)			
Year	Cases	Cantons	Rate[a]	Anomaly (°C)	Cases	Cantons	Rate[a]	Anomaly (°C)
2011	30	5	9.4	+0.11	58	15	1.3	−0.51
2012	24	8	7.1	−0.02	149	22	3.1	+0.12
2013	38	10	9.0	+0.22	123	25	2.6	+0.26
2014	30	7	8.5	−0.10	132	27	2.7	−0.18
2015	50	8	14.1	−0.18	172	29	3.3	+0.11
2016	32	9	8.0	+0.07	105	24	2.0	+0.91
2017	11	4	3.6	+0.74	39	15	0.9	−0.03
2018	22	5	7.4	+0.53	23	9	0.6	+0.55
2019	20	5	5.8	+0.46	101	17	2.0	+0.24
2020	37	6	10.5	+0.50	73	21	1.4	+0.02
2021	35	6	10.1	+0.07	102	20	2.0	−1.21
2022	21	6	6.0	−0.03	87	21	1.7	−1.07
2023	52	7	15.3	−0.15	194	27	3.3	−0.31
2024	398	11	95.7	+0.11	345	23	6.4	−0.49

[a]Dengue hospitalisation rate per 100,000 person-years. “Anomaly” is the average over all canton-month observations within the stratum-year of the deviation between observed monthly mean temperature and the canton-specific 2007–2015 historical baseline. On a rate basis, the low-Andean stratum rose more than 10-fold (9.4 to 95.7 per 100,000) between 2011 and 2024; the corresponding increase in absolute counts (n=30 to n=398) is steeper (13-fold), the difference reflecting population growth. The 2024 rate surge coincided with a record regional dengue epidemic and, notably, with near-zero or negative mean temperature anomalies in both Andean strata, underscoring that the thermal-anomaly association is modest and not the sole driver of that year’s increase (see Table S4). Source: EcoSalud 360; land surface temperature from MODIS (MOD11C3/MYD11C3 V6.1), processed at the parish level using the INEC 2022 administrative cartography (Mendeley Data 10.17632/2yrt5rwnbt.3).

Table S4. Sensitivity analyses for the thermal-anomaly association with dengue hospitalisation risk in Andean cantons (altitude ≥1500 m.a.s.l.). Each row is a separate quasi-Poisson generalised linear model with the canton-month temperature anomaly as exposure (linear and quadratic terms), adjusted for monthly precipitation, NDVI, calendar-month, year and canton fixed effects, with log(population) as offset.

Model specification	Obs.	Cantons	RR at +1 °C vs 0 (95% CI); p
Baseline 2007–2015, incl. 2024 (primary)	12,768	76	1.073 (1.015–1.134); p=0.012
Baseline 2007–2015, excl. 2024	11,856	76	1.040 (1.001–1.080); p=0.047
Non-overlapping baseline 2007–2010, incl. 2024	12,768	76	1.057 (1.017–1.099); p=0.005
Non-overlapping baseline 2007–2010, excl. 2024	11,856	76	1.038 (1.005–1.073); p=0.025

Row 1 is the primary specification reported in the main text. The non-overlapping baseline (2007–2010) removes the temporal overlap between the reference period and the 2011–2024 modelling window; being four years rather than nine, it is a less stable climatological reference and the two baselines should not be read as equally precise. All rows use canton-cluster-robust standard errors, the single variance estimator applied throughout the paper. The association persists under the alternative baseline and attenuates to the margin of conventional significance when the 2024 epidemic year—coincident with a record regional dengue epidemic across the Americas—is excluded (rows 2 and 4). All baselines were computed from the complete MODIS land-surface-temperature series, independently of whether hospitalisations were recorded (Mendeley Data 10.17632/2yrt5rwnbt.3). Source: EcoSalud 360.

Table S5. Residual spatial autocorrelation (global Moran’s I) for the fitted models, Ecuador 2011–2024.

Model	Cantons	Moran’s I	E[I]	p
National model, no canton FE	204	0.143	−0.005	<0.001
National model, with canton FE	204	0.313	−0.005	<0.001
Andean anomaly model, no canton FE	74	0.144	−0.014	0.004
Andean anomaly model, with canton FE (primary)	74	0.034	−0.014	0.179

Global Moran’s I computed on canton-aggregated Pearson residuals using $k = 5$ nearest-neighbour spatial weights derived from canton centroids (INEC 2022 cartography, projected to UTM zone 17S). Cantons whose names recur across provinces were excluded from the spatial join. Moran’s I was computed with the analytic normal approximation (`spdep::moran.test`); k nearest-neighbour weights assign five neighbours to every canton by construction, so no canton is left without neighbours. In the primary Andean model, canton fixed effects reduce residual spatial autocorrelation to a value not distinguishable from zero (I = 0.034; p=0.179). The national pooled model retains appreciable residual spatial structure, and its estimates should be interpreted with that caveat. Source: EcoSalud 360.

Table S6. Cluster-robust inference for the thermal-anomaly model (canton clusters), Andean cantons (altitude ≥1500 m.a.s.l.; 12,768 canton-months; 76 cantons).

Standard errors	RR at +1 °C vs 0	95% CI; p
Model-based (quasi-Poisson)	1.073	1.026–1.122; p=0.002
Canton-cluster-robust (reported throughout)	1.073	1.015–1.134; p=0.012

Cluster-robust standard errors (clustering by canton) computed with the sandwich covariance estimator, applied to the primary canton-fixed-effects model. The point estimate is unchanged and the interval only slightly wider. We adopt the cluster-robust interval as the reported inference throughout the paper—for the primary estimate and for every sensitivity analysis—so that no conclusion depends on which variance estimator is used. Source: EcoSalud 360.

Table S7. Lag-structure selection by quasi-Poisson QAIC (national distributed lag non-linear model with canton fixed effects and canton-wise lagging), Ecuador 2011–2024, and invariance of the minimum hospitalisation temperature to the choice of maximum lag.

Max. lag (months)	Obs.	Effective df	Dispersion	QAIC	MHT (°C)
1	34,112	242	4.27	27,032.9	18.4
2	34,112	242	4.26	27,087.0	18.4
3 (retained, a priori)	34,112	246	4.24	27,152.8	18.4
4	34,112	246	4.23	27,176.1	—

The lagged exposure matrix is constructed *within* each canton, so lagged values never cross canton boundaries; at maximum lag L this removes the first L observations of each of the 208 cantons. All four models are fitted to the identical subset of observations valid at a maximum lag of four months, so their QAIC values are directly comparable.

Two points follow. First, QAIC favours the shortest lag structure examined, which is closer to the biological interval—the extrinsic incubation period of dengue virus in *A. aegypti* (approximately one to two weeks) plus the interval from symptom onset to hospitalisation—than the three-month maximum we specified *a priori*. We retain the three-month structure for consistency with the pre-specified analysis plan and note that it is the more conservative choice. Second, and more importantly, **the estimated minimum hospitalisation temperature is 18.4 °C at every lag structure examined**, so the principal distributed-lag result does not depend on this choice.

The QAIC comparison reported in the previous version of this Supplement was computed with a cross-basis constructed over the stacked canton-month panel, under which lagged values crossed canton boundaries; it has been replaced by the table above. Source: EcoSalud 360.

Table S8. Specification sensitivity of the thermal-anomaly estimate (Andean cantons ≥1500 m.a.s.l.; 12,768 canton-months; 76 cantons). All models include canton fixed effects.

Specification	RR at +1 °C vs 0	95% CI; p
Principal (linear + quadratic anomaly; precipitation, NDVI, month, year and canton fixed effects)	1.073	1.015–1.134; p=0.012
Linear anomaly only (+ covariates)	1.073	1.015–1.136; p=0.014
Temporal and canton fixed effects only (no precipitation/NDVI)	1.070	1.015–1.127; p=0.012
Negative binomial in place of quasi-Poisson (θ = 1.16, estimated by maximum likelihood)	1.049	1.001–1.099; p=0.045

All rows use canton-cluster-robust standard errors. The point estimate is stable across specifications. With canton fixed effects the estimate no longer depends materially on the environmental covariates, in contrast to the specification without canton fixed effects, in which omitting precipitation and NDVI widened the interval to include the null.

The final row addresses the distributional assumption. The Andean analytical set contains a high proportion of zero counts (11,681 of 12,768 canton-months, 91.5%), so the choice between quasi-Poisson and negative binomial is not innocuous. Refitting the primary model as a negative binomial with the dispersion parameter estimated by maximum likelihood attenuates the estimate from 1.073 to 1.049 and widens the interval, but the association remains in the same direction and at nominal significance. We retain quasi-Poisson as the primary specification for consistency with the distributed-lag models and report the negative binomial as a sensitivity analysis. Source: EcoSalud 360.
